# Intracellular production of reactive oxygen species and a DAF-FM-related compound in *Aspergillus fumigatus* in response to antifungal agent exposure

**DOI:** 10.1038/s41598-022-17462-y

**Published:** 2022-08-06

**Authors:** Sayoko Oiki, Ryo Nasuno, Syun-ichi Urayama, Hiroshi Takagi, Daisuke Hagiwara

**Affiliations:** 1 Faculty of Life and Environmental Sciences, University of Tsukuba, 1-1-1 Tennodai, Tsukuba, Ibaraki 305-8577 Japan; 2 Division of Biological Science, Graduate School of Science and Technology, Nara Institute of Science and Technology, 8916-5 Takayama, Ikoma, Nara 630-0192 Japan; 3 Microbiology Research Center for Sustainability, University of Tsukuba, 1-1-1 Tennodai, Tsukuba, Ibaraki 305-8577 Japan; 4 Present Address: Laboratory of Basic and Applied Molecular Biotechnology, Division of Food Science and Biotechnology, Graduate School of Agriculture, Kyoto University, Gokasho, Uji, Kyoto 611-0011 Japan

**Keywords:** Fungal biology, Antifungal agents

## Abstract

Fungi are ubiquitously present in our living environment and are responsible for crop and infectious diseases. Developing new antifungal agents is constantly needed for their effective control. Here, we investigated fungal cellular responses to an array of antifungal compounds, including plant- and bacteria-derived antifungal compounds. The pathogenic fungus *Aspergillus fumigatus* generated reactive oxygen species in its hyphae after exposure to the antifungal compounds thymol, farnesol, citral, nerol, salicylic acid, phenazine-1-carbonic acid, and pyocyanin, as well as under oxidative and high-temperature stress conditions. The production of nitric oxide (NO) was determined using diaminofluorescein-FM diacetate (DAF-FM DA) and occurred in response to antifungal compounds and stress conditions. The application of reactive oxygen species or NO scavengers partly suppressed the inhibitory effects of farnesol on germination. However, NO production was not detected in the hyphae using the Greiss method. An LC/MS analysis also failed to detect DAF-FM-T, a theoretical product derived from DAF-FM DA and NO, in the hyphae after antifungal treatments. Thus, the cellular state after exposure to antifungal agents may be more complex than previously believed, and the role of NO in fungal cells needs to be investigated further.

## Introduction

Controlling pathogenic fungi is a global issue and a long-standing unsolved concern related to crop protection, food security, and public health. Antifungal compounds have been widely used to decrease fungal threats. However, pathogenic fungi that show resistance to certain fungicides and antifungal drugs are being increasingly isolated^1–3^, representing a growing concern in both medical and agricultural areas. Thus, the development of new antifungal agents is needed to control pathogenic fungi and the spread of a potentially harmful mycotoxins produced by these pathogenic fungi.

In general, natural products are main antifungal resources. Many compounds have activities to a wide range of pathogenic fungi, including the causative agents of fungal infection, *Aspergillus fumigatus*, *Candida albicans*, and *Cryptococcus neoformans*. In particular, attention has been paid to essential oils extracted from plants because of their natural and biodegradable properties. Thymol is a major component that is found in the essential oils of *Monarda punctata*^4^ and *Thymus kotschanus*^5^, which have moderate antifungal activities (200 to 350 µg/mL) against *Aspergillus niger*, *Aspergillus ochraceus*, *Alternaria alternata*, and *Fusarium oxysporum*^6^. In another study, thymol had a minimal inhibitory concentration (MIC) of 80 µg/mL against *Aspergillus flavus*^7^. Farnesol is a natural sesquiterpene alcohol that is a composite of *Matricaria recutita* flower essential oil^8^ and *Litsea coreana* leaf essential oil^9^. This compound acts as a quorum-sensing molecule in *C. albicans* and is involved in the inhibition of the yeast-to-hypha transition^10^, thereby blocking biofilm formation^11^. It has been linked to significant fungal growth alterations, such as conidiation inhibition, growth retardation, and apoptosis-like cell death, in several species of pathogenic fungi^12–15^.

The antibacterial effects of essential oils result from targeting cell walls and cytoplasmic membranes, which leads to the leakage of essential ions, decreases in membrane potential, and the dysfunction of membrane proteins^16,17^. In contrast, the mechanisms underlying antifungal activities of essential oils are largely unknown. Several pathogenic fungi generate reactive oxygen species (ROS) in response to the medically used antifungals itraconazole, terbinafine, and amphotericin B^18^, as well as thymol and farnesol^7,19^. Shen et al.^7^ also demonstrated that thymol facilitates nitric oxide (NO) generation in the spores of *A. flavus*, which contributes to the fungicidal activity. These cellular responses to antifungal agents are clues to understanding the target mechanisms, which consequently aid in the development of novel antifungal drugs. In this study, we investigated the cellular responses to several natural products and medically used antifungals in the pathogenic fungus *Aspergillus fumigatus* by determining ROS and NO contents in the hyphae.

## Results

### Intracellular production of ROS in response to antifungal agents

**Figure 1:**
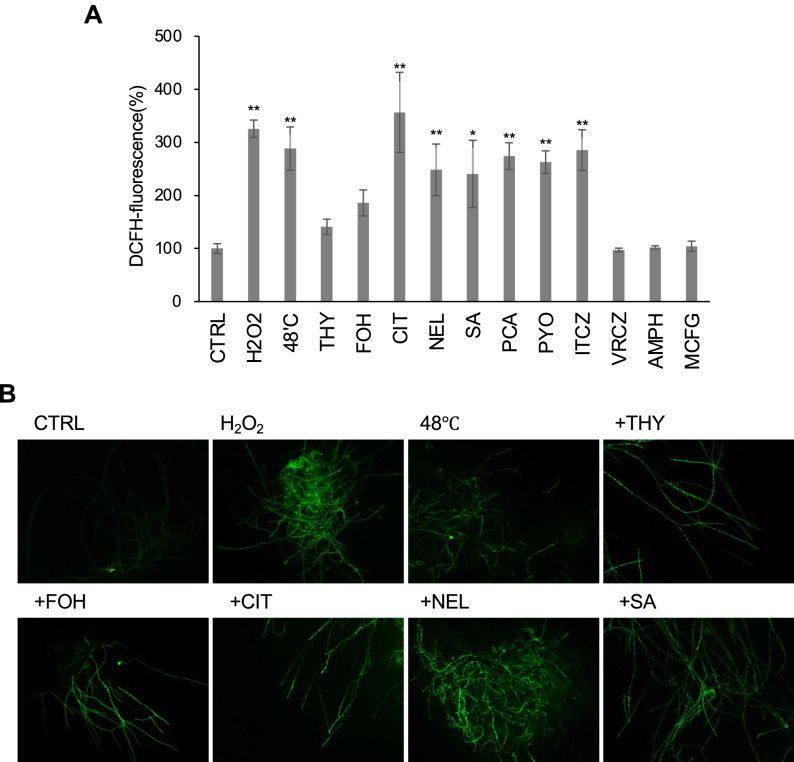
ROS production in *A. fumigatus* hyphae in response to antifungal treatments. (**A**) Intracellular ROS production, as indicated by DCFH-DA, was quantified. CTRL indicates the control sample without any stressors. The fluorescence intensity per protein amount of CTRL was defined as 100%. The cells were disrupted, and the extracts were used for fluorescence intensity measurement. The values were obtained from three replicates, and the error bar represents the standard deviation. H2O2: 10 mM hydrogen peroxide, THY: 1 mM thymol, FOH: 1 mM farnesol, CIT: 1 mM citral, NEL: 1 mM nerol, SA: 1 mM salicylic acid, PCA: 1 mM phenazine-1-carboxylicacid, PYO: 1 mM pyocyanin, ITCZ: 10 µg/mL itraconazole, VCZ: 10 µg/mL voriconazole, AMPH: 10 µg/mL amphotericin B, and MCFG: 10 µg/mL micafungin. Significant differences compared with the CTRL sample were examined using Dunnett’s tests and are indicated as *p < 0.05; **p < 0.01. (**B**) Intracellular ROS was visualised using DCFH-DA and a fluorescence microscope. The cells were treated in the same way for the fluorescence intensity measurement.

The production of ROS is caused under physiological conditions in all cells, but ROS are toxic only when they are generated to high levels and oxidative stress takes place^20^. Here, we selected the life-threatening pathogenic fungus *A. fumigatus* as a model because treating its infection is challenging owing to the limited number of approved drugs. First, the hyphae of *A. fumigatus* were treated with hydrogen peroxide as an oxidative stress and transferred to high-temperature condition as a temperature stress. Under these conditions, ROS levels detected using 2′,7′-dichlorofluorescein diacetate (DCFH-DA) were elevated as determined by a fluorescent microscope and plate reader, suggesting that ROS were generated during adaptation to these stresses (Fig. 1A,B). Then, we investigated whether *A. fumigatus* intracellularly generated ROS after exposure to antifungal agents. To increase our understanding, a wide variety of natural products, including plant-derived and bacteria-producing compounds, as well as medical antifungal agents, were used. When *A. fumigatus* mycelia were treated with citral, nerol, and the plant hormone salicylic acid (SA), ROS were produced significantly in the hyphae, as detected by DCFH-fluorescence (Fig. 1A,B). Although no significant increase of DCFH-fluorescence was observed in the cell lysates, a clear fluorescent signal could be detected in the hyphae under a fluorescent microscope after treatment with thymol and farnesol (Fig. 1B). Exposure to phenazine-1-carbonic acid (PCA) and pyocyanin also resulted in elevated levels of ROS production (Fig. 1A), whereas no production of ROS was detected after exposure to pyrrolnitrin (data not shown). In response to itraconazole, but not to voriconazole, amphotericin B, and micafungin, ROS production occurred (Fig. 1A). Antifungal compounds showed no effect on DCFH DA fluorescence outside the cell system (Supplementary Fig. S1). These results suggested that a wide range of antifungal compounds facilitated ROS production in this fungus.

### Detection of reactive nitrogen species (RNS) in response to antifungal agents

**Figure 2:**
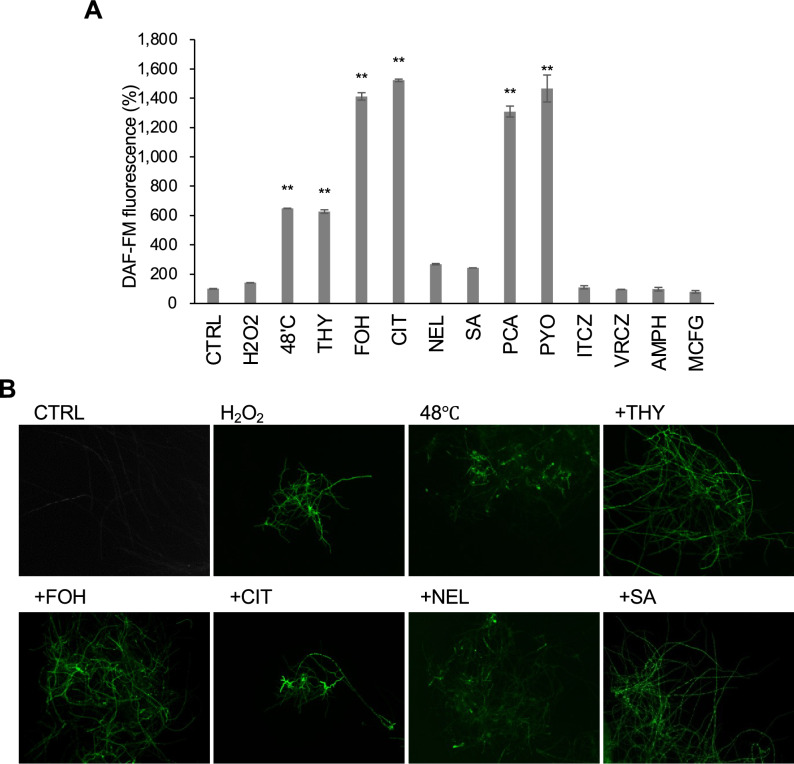
NO production in *A. fumigatus* hyphae in response to antifungal treatments. (**A**) Intracellular NO production, as indicated by DAF-FM DA, was quantified. CTRL indicates the control sample without any stressors. The fluorescence intensity per protein amount of CTRL was defined as 100%. The cells were disrupted, and the extracts were used for fluorescence intensity measurement. The value was obtained from three replicates, and the error bar represents the standard deviation. H2O2: 10 mM hydrogen peroxide, THY: 1 mM thymol, FOH: 1 mM farnesol, CIT: 1 mM citral, NEL: 1 mM nerol, SA: 1 mM salicylic acid, PCA: 1 mM phenazine-1-carboxylicacid, PYO: 1 mM pyocyanin, ITCZ: 10 µg/mL itraconazole, VCZ: 10 µg/mL voriconazole, AMPH: 10 µg/mL amphotericin B, and MCFG: 10 µg/mL micafungin. Significant differences compared with the CTRL sample were examined using Dunnett’s tests and are indicated as *p < 0.05; **p < 0.01. (**B**) Intracellular NO was visualised using DAF-FM DA and a fluorescence microscope. The cells were treated in the same way for the fluorescence intensity measurement.

As a cellular response to abiotic stresses, the generation of RNS has been reported in multiple fungal species^7,21,22^. The intracellular production of NO has been conventionally detected using diaminofluorescein-FM diacetate (DAF-FM-DA). We investigated whether *A. fumigatus* produced NO in response to the antifungal agents using DAF-FM DA. First, we examined the *A. fumigatus* hyphae after oxidative and high-temperature stresses. The levels of detected fluorescence suggested that the intracellular NO level increased at high temperature (Fig. 2A). This increased signal level was also observed in hyphae treated with thymol, farnesol, citral, PCA, and pyocyanin (Fig. 2A). However, treatment with the antifungal drugs did not result in NO production (Fig. 2A). Observations under a fluorescent microscope using DAF-FM DA revealed that the signals appeared in *A. fumigatus* hyphae in response to thymol, farnesol, citral, nerol, and salicylic acid, as well as oxidative (hydrogen peroxide) and high-temperature (48 °C) stresses (Fig. 2B). Antifungal compounds showed no effect on DAF-FM DA fluorescence outside the cell system (Supplementary Fig. S1). These results suggested that NO was produced in *A. fumigatus* hyphae after exposure to plant-derived antifungal compounds. Notably, the fluorescent signals after farnesol exposure appeared in media containing nitrate, ammonium sulphate, or proline as the sole nitrogen source, indicating that intracellular NO production was independent of the culture’s nitrogen source (Supplementary Fig. S2).

### Potential associations of ROS and RNS productions with growth inhibition

**Figure 3:**
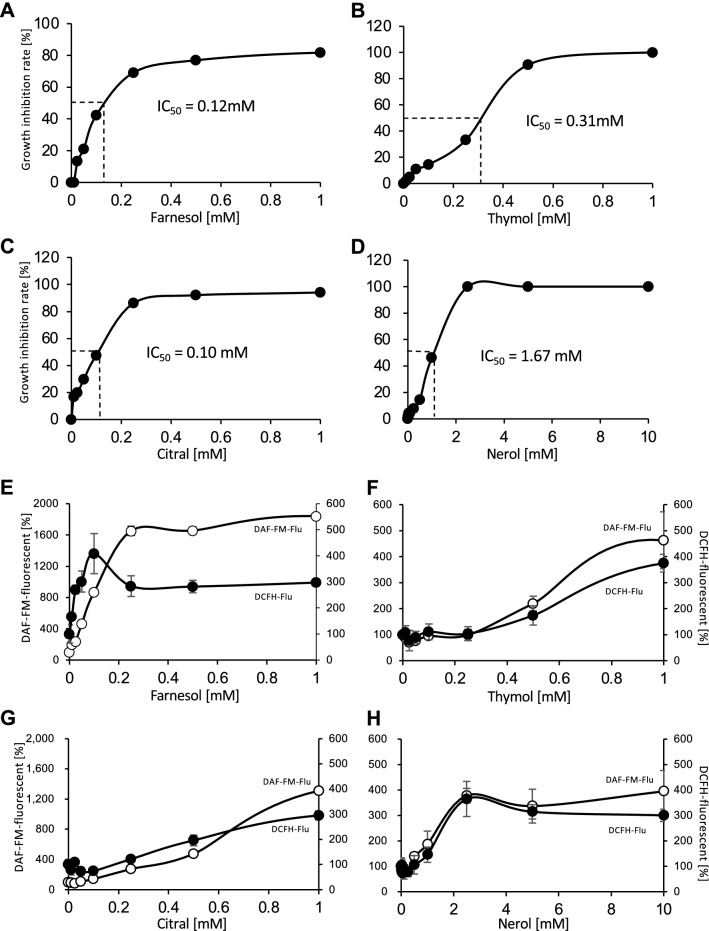
Relationships between antifungal compound concentrations and the growth inhibitory effects or productions of DCFH- and DAF-FM-related fluorescence. The growth inhibition rates were determined after treatment with different concentrations of farnesol, thymol, citral, and nerol (**A**–**D**, respectively). The calculated IC_50_ values are depicted in the graph. The induced DCFH- and DAF-FM-related fluorescence intensity levels, shown by black and white circles, respectively, were measured after treatment with farnesol, thymol, citral, and nerol (**E**–**H**, respectively).

To gain more insight into the effects of ROS and NO on the antifungal activities of the plant-derived compounds, IC_50_ values were determined (Fig. 3A–D). The IC_50_ values for farnesol, thymol, citral, and nerol were 0.12 mM, 0.31 mM, 0.10 mM, and 1.67 mM, respectively. The intensity levels of the DCFH-fluorescence and DAF-FM-fluorescence were determined for different concentrations of the antifungal agents (Fig. 3E–H). After farnesol treatment, DCFH-fluorescence showed a maximum intensity near the IC_50_ concentration, whereas DAF-FM-fluorescence reached a maximum at a concentration that was twice the IC_50_ value. At this value, 70% of the fungal growth was inhibited (Fig. 3E). We conducted multiple regression analysis to investigate correlation between growth inhibition, concentrations of antifungal agents, ROS level, and NO level. As a result, some correlations were observed as follows: between growth inhibition by farnesol and NO level (*P* = 0.0053), growth inhibition by thymol and concentration of thymol (*P* = 0.035), growth inhibition by citral and concentration of citral (*P* = 0.0052), growth inhibition by citral and NO level (*P* = 0.017), growth inhibition by nerol and concentration of nerol (*P* = 0.024) (Supplementary Fig. S3). Consequently, we focused on farnesol exposure to determine whether the elevated intracellular ROS and NO levels were related to growth inhibition.

**Figure 4:**
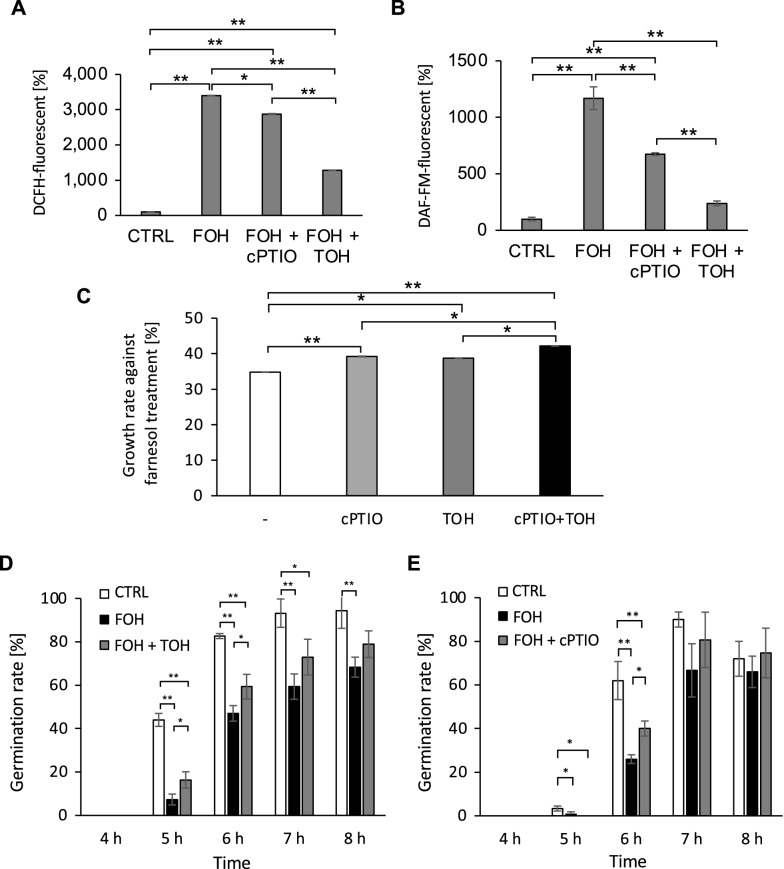
The effects of ROS and NO scavengers. (**A**) The DCFH-related fluorescence intensity after treatment with farnesol was measured with or without cPTIO and tocopherol (TOH). (**B**) The DAF-FM-related fluorescence intensity after treatment with farnesol was measured with or without cPTIO and TOH. The data were obtained from three biological replicates, and the error bar represents the standard deviation. (**C**) The effects of the scavenger compounds on farnesol-induced growth inhibition were evaluated. (**D**) The effects of TOH on germination kinetics in the presence of farnesol. (**E**) The effects of cPTIO on germination kinetics in the presence of farnesol. The data were obtained from three biological replicates, and the error bar represents the standard deviation. Significant difference between samples were examined using Tukey’s tests and are indicated as *p < 0.05; **p < 0.01.

Tocopherol and carboxy-PTIO (cPTIO) are widely used as antioxidant and NO scavengers, respectively. In the presence of tocopherol, the intracellular ROS content, which was elevated by the farnesol treatment, decreased (Fig. 4A). Similarly, exposure to cPTIO resulted in a lowered intracellular DAF-FM-fluorescence intensity compared with in farnesol-treated cells (Fig. 4B). Thus, scavenging compound treatments were at least partly able to eliminate ROS and NO from the cells. Consequently, we investigated whether tocopherol and cPTIO affect colony growth on PDA supplemented with farnesol. Tocopherol and cPTIO weakened the growth inhibitory effects of farnesol (Fig. 4C). The conidia of *A. fumigatus* started germinating at 5 h in Potato Dextrose Broth (PDB). The germination rate was significantly lower in the presence of farnesol. When supplied with tocopherol, the germination rate was suppressed at 5 h and 6 h (Fig. 4D). This observation suggested that the elimination of intracellular ROS resulted in the recovery of germination in *A. fumigatus* conidia. In the presence of cPTIO, the lowered germination rate resulting from farnesol exposure was also suppressed at 6 h (Fig. 4E). This suggested that the inhibition of germination caused by farnesol was at least partly related to intracellular NO production.

### Intracellular NO measured using the Griess method

**Figure 5:**
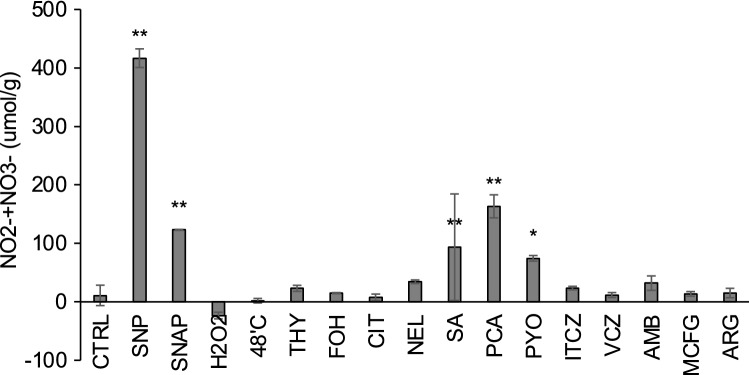
Measurement of the intracellular NO level using the Griess method. *Aspergillus fumigatus* was grown in GMM(Pro) and treated with the shown stressors. Each extract was obtained as described for the quantification of DCFH- or DAF-FM-related fluorescence. An aliquot was used for protein quantification, and proteins were removed in the rest of cell extracts. To determine the NO level of the sample, absorbance at 540 nm was quantified using an NO_2_/NO_3_ Assay Kit-C II (Colorimetric). The signal intensity was calculated in accordance with the instructions. For the L-arginine supplementation, grown cells were treated with 10 mM L-arginine. The data were obtained from three biological replicates, and the error bar represents the standard deviation. Significant differences between samples were examined using Dunnett’s tests and are indicated as *p < 0.05; **p < 0.01.

In these experiments, the intensity of DAF-FM fluorescence decreased significantly in the presence of farnesol plus tocopherol compared with farnesol alone (Fig. 4B). This result raised the question of whether the DAF-FM signal was affected by the ROS level. Another possibility was that DAF-FM fluorescence occurred in the presence of ROS but not NO. To address this question, NO homeostasis in the hyphae was investigated using the Griess method to determine the NO content. When treated with oxidative and high-temperature stresses, in contrast to our expectation, there was no significant increased level of NO production (Fig. 5). After treatment with other antifungal agents including farnesol and thymol, no significant NO production was detected, except after salicylic acid, PCA, and pyocyanin treatments (Fig. 5). Thus, NO did not appear to be produced in the *A. fumigatus* hyphae treated with antifungal agents.

### Accumulations of novel DAF-FM-related fluorescent signals in the hyphae

**Figure 6:**
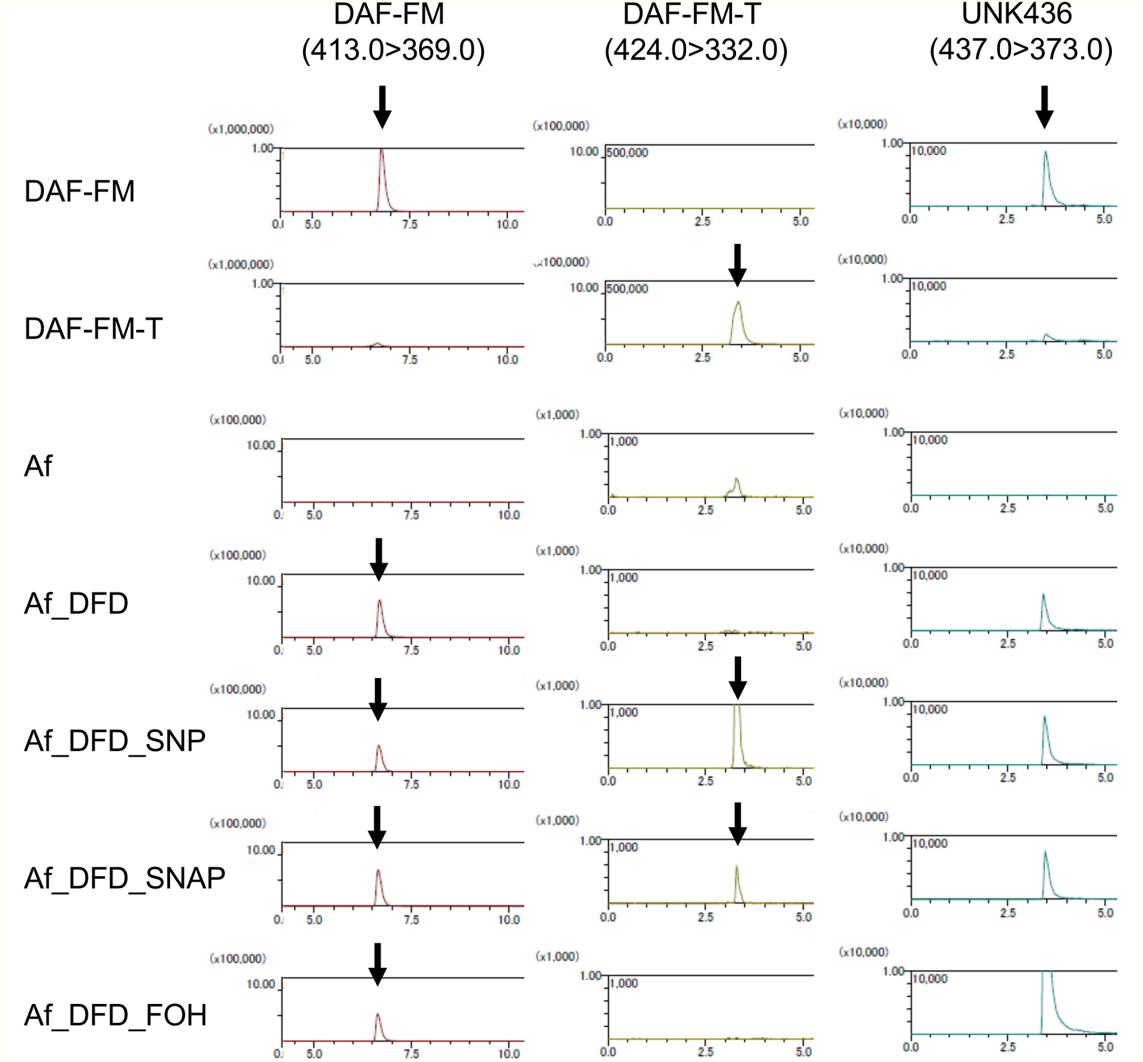
Detection of DAF-FM-T and a DAF-FM-related unknown product. *Aspergillus fumigatus* was grown in PDB, then supplemented with or without DAF-FM DA (Af or Af_DFD, respectively). DAF-FM DA-treated cells were incubated with sodium nitroprusside (SNP) or S-nitroso-N-acetylpenicillamine (SNAP) as NO donors (Af_DFD_SNP or Af_DFD_SNAP, respectively). DAF-FM-DA-treated cells were incubated with farnesol (Af_DFD_FOH). Each extract was obtained as described for the quantification of DCFH- or DAF-FM-related fluorescence and analysed by LC–MS/MS. DAF-FM was detected with fragments of *m/z* 413.0 > 369.0, whereas DAF-FM-T was 424.0 > 332.0. The unknown product (UNK436) was detected as the fragment 437.0 > 373.0. As a standard, DAF-FM was incubated with or without SNAP in 10 mM Tris–HCl (pH 7.9) at 37 °C for 60 min (DAF-FM-T or DAF-FM, respectively).

Because of the controversial NO production results, we wanted to clarify whether the DAF-FM fluorescence signal was derived from a reaction with NO. It has been reported that DAF-FM can directly react with NO molecules, resulting in diaminofluorescein FM triazole (DAF-FM-T), which is a fluorescent compound with a molecular weight of 423 kDa^23^. Thus, a tandem mass spectrometry (LC–MS/MS) analysis was performed to determine whether DAF-FM-T was produced in the hyphae in response to the antifungal treatments. As a control, DAF-FM-T production was successfully confirmed using the fragmentation trait of 437 > 332 in *A. fumigatus* hyphae treated independently with the NO donors sodium nitroprusside and S-nitroso-N-acetylpenicillamine (Fig. 6). However, treatment with farnesol did not result in DAF-FM-T production (Fig. 6). This was also true under both oxidative- and high-temperature-stress conditions (Supplementary Fig. S4). Moreover, *A. fumigatus* did not produce DAF-FM-T after independent treatments with thymol, citral, nerol, salicylic acid, PCA, pyocyanin, itraconazole, voriconazole, amphotericin B, and micafungin (Supplementary Fig. S4). Thus, NO production in *A. fumigatus* hyphae was highly limited in response to the stressors. However, an unknown product with *m/z* of 437 (UNK436) was spontaneously produced in the presence of DAF-FM in the culture independent of fungal inoculation (Fig. 6). Notably, the UNK436 production appeared to increase in response to farnesol treatment (Fig. 6: Af_DFD vs Af_DFD_FOH). NO was not observed in this experiment likely because the main route of its production is via reductive pathway from nitrate^24^.

**Figure 7:**
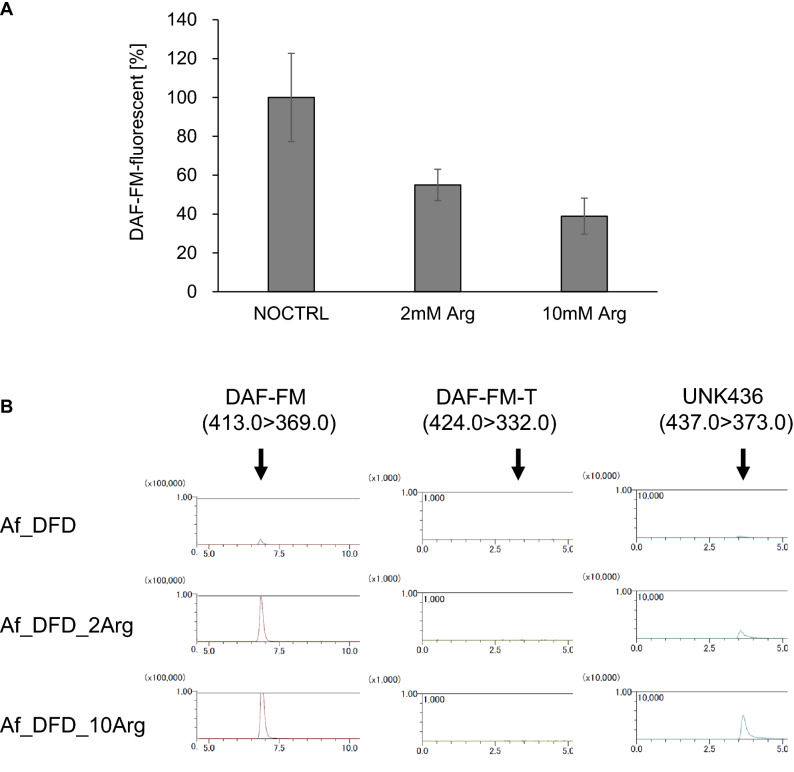
Detection of a DAF-FM-related product in medium supplemented with L-arginine. (**A**) *Aspergillus fumigatus* grown in PDB was treated with DAF-FM DA, followed by incubation with 0 mM, 2 mM, or 10 mM L-arginine (Af_DFD, Af_DFD_2Arg, or Af_DFD_10Arg, respectively). Each extract was obtained, and the DAF-FM fluorescence was quantified, as described previously. (**B**) DAF-FM, DAF-FM-T, and UNK436 were detected by the LC–MS/MS analysis.

In mammals and bacteria, NO is synthesised from L-arginine and oxygen by the catalytic activity of NO synthases^25,26^. Although NO biosynthesis and functions in fungi have not been thoroughly explored, *Aspergillus nidulans*, a close relative of *A. fumigatus*, has been reported recently to provoke NO production in the presence of L-arginine^27^. In accordance with the literature, we used DAF-FM fluorescence quantification (Fig. 7A), the Griess method (Fig. 5), and DAF-FM-T detection (Fig. 7B) to determine whether *A. fumigatus* produces NO in the presence of L-arginine. In contrast to our expectation, the presence of L-arginine did not increase the NO level in *A. fumigatus* hyphae as assessed by all three detection systems.

## Discussion

Numerous natural compounds have been isolated as antifungal agents from biological resources, such as plants and microorganisms, through extensive screening. The modes of action for the reported compounds remain largely unknown because defining the molecular targets is time-consuming and difficult work. A better understanding of the cellular responses to antifungal agents and stressed states will aid in developing efficient screening systems or applications of new drugs.

*Aspergillus fumigatus* is a major pathogenic fungus of humans, causing life-threatening mycoses. Owing to the limited available antifungal agents, the development of new drugs is an urgent issue. The present study examined the effects of many antifungal compounds against *A. fumigatus* and demonstrated that most of the antifungals tested triggered ROS production in the hyphae. Typical intracellular ROS includes superoxide and hydrogen peroxide, which are formed during cellular metabolism and energy conversion at a certain level. The intracellular production of ROS is a major and well-known response to antifungal agents in fungal cells. Mitochondria are the principal generators of intracellular ROS. In fact, amphotericin B induces ROS production in a mitochondria-dependent manner in several pathogenic yeast including *C. albicans* and *C. neoformans*^28^. Additionally, itraconazole and amphotericin B increase the intracellular ROS level in *A. fumigatus*^18^. The addition of a complex I inhibitor, rotenone, reduces ROS production and causes a lower susceptibility to the drugs, indicating that the intracellular ROS is at least partly responsible for the antifungal activity. In line with the previous report, an antioxidant compound, tocopherol, reduced the farnesol-induced ROS level, which suggested that oxidative stress intracellularly causes growth inhibition. Notably, one of the compounds tested, the plant hormone SA, triggered ROS production in the hyphae of *A. fumigatus*. To our knowledge this is the first report of a fungal response to SA exposure. The antifungal effects of SA itself have been reported, in which the growth and germination of *Fusarium graminearum* were inhibited by less than 0.8 mM SA^29^. The growth of *A. flavus* is affected by 1 mM SA and completely inhibited by the addition of 9 mM SA to PDA medium^30^. Here, 1 mM SA inhibited 20% of *A. fumigatus* growth and 5 mM completely blocked growth (data not shown); therefore, ROS production may have a growth inhibitory effect against *A. fumigatus*.

Another oxidative agent is RNS, which mostly originates from NO. NO can react with ROS, usually in the form of superoxide, to produce peroxynitrite (ONOO-)^31^, which causes strong nitrosative stresses, such as lipid peroxidation in cells and organelle membranes, the oxidation and nitration of proteins, DNA damage, and even the initiation of apoptosis^32^. Exogenous NO induces sexual development and decreases secondary metabolite production in *A. nidulans*^33^. These findings suggested a regulatory role for NO during fungal developmental stages. Endogenous NO has been analysed in several fungi, including various plant pathogens, as well as *A. nidulans*. By staining with DAF2-DA, fluorescent signals of NO production have been detected in hyphae of *B. cinerea* growing on a solid medium^34^. *Neurospora crassa* has also been shown to produce NO in liquid culture at the early vegetative stage and during conidiation^35^. Marcos et al. demonstrated that the fluorescent signal of NO production increases in a light-dependent manner in *A. nidulans*^22^. The add ition of L-arginine to the media results in NO production in the hyphae^27^. Consequently, we expected *A. fumigatus* to produce NO in response to environmental stress. Indeed, using DAF-FM-DA, clear signals were detected in the hyphae after independent treatments with farnesol, thymol, citral, nerol, and SA, as well as high-temperature and oxidative stresses (Fig. 2a,b). Regression analysis revealed the correlation between NO production and growth inhibition by farnesol and citral. In contrast to our expectation, however, the Griess method and an LC–MS/MS analysis did not support the results. These data strongly suggested that NO was not produced in the hyphae, and another unknown molecule reacted with DAF-FM resulting in a fluorescent signal. Supplementation with L-arginine did not induce NO production, which indicated there were different mechanisms underlying NO homeostasis in *A. nidulans* and *A. fumigatus* even though they belong to same genus. The differential metabolic responses to the stresses may contribute to the adaptability to in-host environments during infection. NO homeostasis in pathogenic fungi will be a focus of a future study.

DAF-FM and the related compounds have been widely used for detecting NO in tissues and cells of various organisms. Most fungal studies used DAF-FM to detect NO^22,27,33–35^. In this study, however, we provided a clear example in which DAF-FM DA reacted with the other molecule, not NO. Indeed, DAF-FM has been suggested to react with N_2_O_3_ as well as NO to give a fluorescent product in complex biological systems^36,37^. Thus, additional experiments are necessary to clarify the results and draw a conclusion. Germination assays after farnesol treatment were performed using the NO scavenger cPTIO. Although the Griess method and LC–MS/MS analyses determined that there was no production, cPTIO reduced the farnesol-induced DAF-FM-fluorescence and suppressed the farnesol-dependent growth inhibition and germination delay (Fig. 4B,C,E). There may be unknown mechanisms behind the farnesol actions affected by cPTIO supplementation. One possible explanation for that is the reduction in intracellular ROS by cPTIO as the DCFH-fluorescence was slightly but significantly reduced by the application of cPTIO (Fig. 4A). Further experiments are required to determine whether DAF-FM DA can react with ROS or can be activated when the intracellular ROS level increases.

In conclusion, intracellular ROS was produced when the fungal hyphae were challenged with lethal antifungal reagents. Several antifungal agents, including SA, were shown for the first time to induce ROS generation. The intracellular oxidative stress agents may be related to fungicidal activity. We need to further expand our knowledge of intracellular ROS and RNS in fungi to establish efficient and broad-based drug-screening methods.

## Materials and methods

### Strain and growth media

*Aspergillus fumigatus* strain Af293 was used in this study. The strain was cultured in PDB at 37 °C on a rotary shaker at 120 rpm throughout the study. In some experiments, glucose minimal medium (GMM) (1%(w/v) glucose, 0.052% KCl, 0.052% MgSO_4_·7H_2_O, 0.152% KH_2_PO_4_, 0.0022% ZnSO_4_·7H_2_O, 0.0011% H_3_BO_3_, 0.0005% MnCl_2_·4H_2_O, 0.0005% FeSO_4_·7H_2_O, 0.00016% CoCl_2_·5H_2_O, 0.00016% CuSO_4_·5H_2_O, 0.00011% (NH_4_)6Mo_7_O_24_·4H_2_O, 0.005% Na_4_EDTA, pH 6.5) containing variable nitrogen sources, such as sodium nitrate, ammonium sulphate, and proline (70 mM) were used, which were designated GMM(NO_3_), GMM(NH_4_), and GMM(Pro), respectively. The antifungal chemicals used were commercially obtained, as follows: thymol (NACALAI TESQUE, Inc., Kyoto, Japan), farnesol (Sigma-Aldrich Co., St. Louis, MO, USA), citral (NACALAI TESQUE, Inc.), nerol (Tokyo Chemical Industry Co., Ltd, Tokyo, Japan), salicylic acid (NACALAI TESQUE, Inc.), PCA (Wako Pure Chemicals Industries, Osaka, Japan), pyocyanin (Cayman Chemical Company, Ann Arbor, MI, USA), itraconazole (Wako Pure Chemicals Industries), voriconazole (Tokyo Chemical Industry Co., Ltd), and amphotericin B (Sigma-Aldrich Co.). Micafungin was a generous gift from Dr. Keietsu Abe, Tohoku University.

### ROS assay

Intracellular ROS was visualised using DCFH-DA and an inverted fluorescence microscope (excitation 488 nm and emission 525 nm) (Axio, Zeiss, Oberkochen, Germany). To quantify ROS levels, fungal cells were grown in PDB for 18 h, and 2.5 µg/mL DCFH-DA was added to the culture, followed by incubation at 37 °C for 30 min in the dark. Cells were exposed to various stress factors, such as 10 mM hydrogen peroxide, 1 mM thymol, 1 mM farnesol, 1 mM citral, 1 mM nerol, 1 mM salicylic acid, 1 mM PCA, 1 mM pyocyanin, 10 µg/mL itraconazole, 10 µg/mL voriconazole, 10 µg/mL amphotericin B, and 10 µg/mL micafungin, at 37 °C for 30 min. Cells were then washed three times with sterilised water. As a high-temperature stress, the culture was incubated at 48 °C for 30 min. The cell suspension was disrupted by a FastPrep-24 (MP Biomedicals) with glass beads at 4 m/s for 20 s and centrifuged at 12,000 *g* for 10 min. Fluorescence intensity was measured in the supernatants of cell extracts using a 96-well plate reader system as follows: excitation length, 485/20; emission length, 528/20; optics, top; read speed, normal; delay, 100 ms; and read height, 1 mm. The protein concentrations were measured using a Qubit™ Protein Assay Kit (Thermo Fisher Scientific). The fluorescence intensity per protein amount was calculated for each sample, and these were compared with that of the control treated with no stress.

### NO assays

Intracellular NO was visualised using DAF-FM DA (Goryo Chemical Inc., Sapporo, Japan). The quantification of the intracellular NO level using DAF-FM DA was performed in a manner comparable to the ROS quantification described above. In brief, the cells were harvested after a 30-min DAF-FM-DA treatment, and the suspension was disrupted. Fluorescence intensity was measured in the supernatants of cell extracts using a 96-well plate reader system (Excitation, 485/20; Emission, 528/20).

### Growth test

In total, 1 µL of an *A. fumigatus* spore suspension (10^7^/mL) was inoculated on PDA in the presence or absence of 1 mM farnesol plus 0.1 mM cPTIO (Dojindo, Kumamoto, Japan) and 0.1 mM tocopherol (NACALAI TESQUE, Inc.). After incubation at 37 °C for 3 days, the diameters of colonies were measured. The IC_50_ values were calculated using KaleidaGraph (Synergy Software, Reading, PA).

### Germination test

The spores of *A. fumigatus* (10^4^) were inoculated in PDB and incubated at 37 °C at 120 rpm in the presence or absence of 1 mM farnesol and 0.1 mM tocopherol. Aliquots of the culture were collected every hour. When a germ tube was generated from a swelled spore, the spore was considered to have germinated. The germination rate for each sample was determined using a light microscope, with three biological replicates.

### Griess method for NO detection

Fungal cells were grown in GMM(Pro) medium, and the stress-treated cell extracts were obtained using the same method described above. After quantifying the amounts of the proteins, the proteins were removed from the cell extracts using Amicon Ultra 0.5-mL filters (Merck, Darmstadt, Germany). The resultant samples were transferred to a 96-well plate, and the NO level was quantified using an NO_2_/NO_3_ Assay Kit-C II(Colorimetric) ~ Griess Reagent Kit ~ (Dojindo) at 540 nm.

### Chemical detection by LC–MS/MS

To detect DAF-FM-T, a theoretical product of NO and DAF-FM, in the DAF-FM DA-treated cells, an LC–MS/MS analysis was performed. The cell extracts were obtained as described above. Methanol was added to each cell extract to produce a 60% final concentration. The cell extracts were filtered and centrifuged at 12,000 *g* for 5 min to remove proteins, and the supernatant was used as the LC–MS/MS sample. LC–MS/MS analysis using an Acquity UPLC BEH C18 1.7-µm column (Waters, Milford, MA) was performed with an LCMS™-8045 (Shimadzu Corp., Kyoto, Japan). A gradient elution of 5–100% acetonitrile was used as the mobile phase, with a flow rate of 0.3 mL/min. The positive electrospray ionization mode was selected with fragments of *m/z* 413.0 > 369.0 (DAF-FM), 424.0 > 332.0 (DAF-FM-T), and 437.0 > 373.0 (UNK436). The standard DAF-FM-T was prepared in vitro as described previously using DAF-FM (Goryo Chemical Inc.) and a NO-donor^38^.

## Supplementary Information

Supplementary Information.

## Data Availability

All data generated or analyzed during this study are included in this published article and its supplementary information files.
