# Supplementary material for: Intracellular production of reactive oxygen species and a DAF-FM-related compound in Aspergillus fumigatus in response to antifungal agent exposure
Source: Sci Rep. 2022 Aug 6;12:13516. doi: 10.1038/s41598-022-17462-y (PMC9357077; doi:10.1038/s41598-022-17462-y)
Supplement: Supplementary file 1 — Supplementary Information. [file 41598_2022_17462_MOESM1_ESM.pdf]

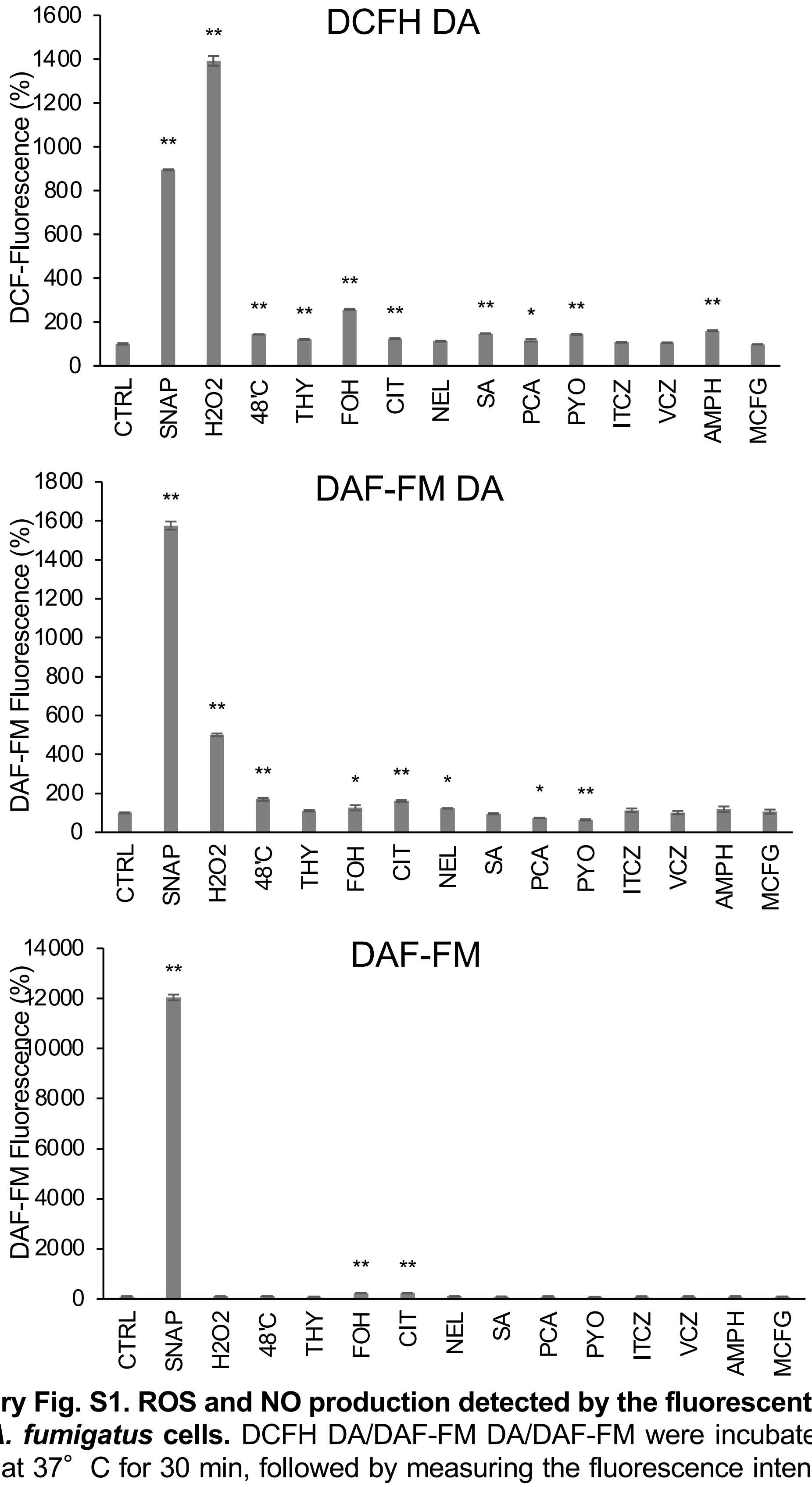

Supplementary Fig. S1. ROS and NO production detected by the fluorescent probes in the absence of *A. fumigatus* cells. DCFH DA/DAF-FM DA/DAF-FM were incubated with various stress factors at 37° C for 30 min, followed by measuring the fluorescence intensity. The value was obtained from three replicates, and the error bar represents the standard deviation. Significant differences between samples were examined using Dunnett's tests and are indicated as *: p < 0.05; **: p < 0.01. H2O2: 10 mM hydrogen peroxide, THY: 1 mM thymol, FOH: 1 mM farnesol, CIT: 1 mM citral, NEL: 1 mM nerol, SA: 1 mM salicylic acid, PCA: 1 mM phenazine-1-carboxylic acid, PYO: 1 mM pyocyanin, ITCZ: 10 µg/mL itraconazole, VCZ: 10 µg/mL voriconazole, AMPH: 10 µg/mL amphotericin B, and MCFG: 10 µg/mL micafungin. Significant differences compared with the CTRL sample were examined using Dunnett's test and are indicated as *: $p < 0.05$; **: $p < 0.01$.

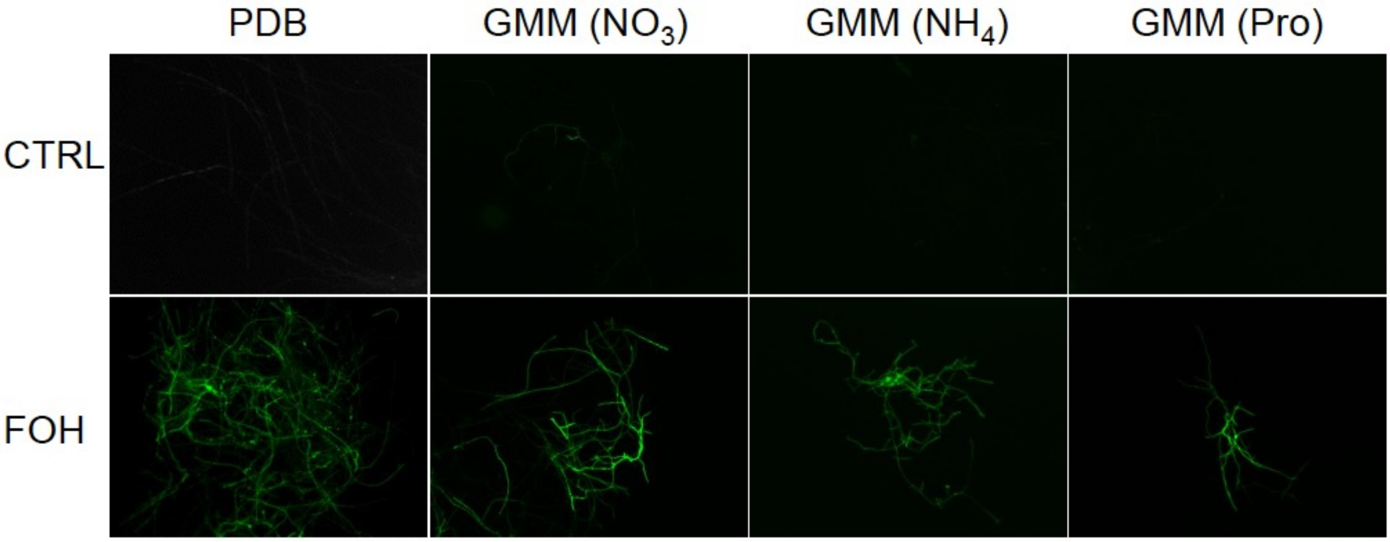

Supplementary Fig. S2. Visualization of NO in *A. fumigatus* hyphae cultured in media containing nitrate, ammonium, or proline as the sole nitrogen source. *A. fumigatus* cells were cultured in potato dextrose broth (PDB), or glucose minimal medium (GMM) containing nitrate (NO_3), ammonium sulphate (NH_4), or proline (Pro). DAF-FM DA-treated hyphae were incubated with farnesol and observed with a fluorescence microscope.

Growth inhibition by farnesol

variable	Correlation coefficient	Regression coefficient	F	T	P-value	Regression equation	Lower 95%	Upper 95%
concentration of FOH				0.60	0.58	y=4.72x-10.38	-17.03	26.46
ROS level	0.996	0.993	0.0000979	2.17	0.096	y=0.040x-10.38	-0.01	0.09
NO level				10.17	0.0053	y=0.042x-10.38	0.03	0.05

Growth inhibition by thymol

variable	Correlation coefficient	Regression coefficient	F	T	P-value	Regression equation	Lower 95%	Upper 95%
concentration of THY				3.13	0.035	y=162.43x+41.78	-0.60	1.50
ROS level	0.981084	0.963	0.00259	-1.93	0.13	y=-0.82x+41.78	-2.01	0.36
NO level				1.19	0.30	y=0.45x+41.78	18.22	306.64

Growth inhibition by citral

variable	Correlation coefficient	Regression coefficient	F	T	P-value	Regression equation	Lower 95%	Upper 95%
Concentration of CIT				5.54	0.0052	y=453.65x+72.32	226.20	681.09
ROS level	0.972724	0.946	0.00533	-1.90	0.13	y=-0.47x+72.32	-1.15	0.22
NO level				-3.93	0.017	y=-0.22x+72.32	-0.38	-0.07

Growth inhibition by nerol

variable	Correlation coefficient	Regression coefficient	F	T	P-value	Regression equation	Lower 95%	Upper 95%
concentration of NEL				3.538	0.024	y=39.33x-4.96	8.468	70.198
ROS level	0.977	0.955	0.00373	0.407	0.705	y=0.055x-4.96	-0.318	0.427
NO level				0.019	0.986	y=0.0024x-4.96	-0.337	0.342

Supplementary Fig. S3. Regression analysis between growth inhibition by antifungals and concentrations/ROS level/NO level. Growth inhibition was set as target variable. Concentrations of antifungals/ROS level/NO level were set as explanatory variable.

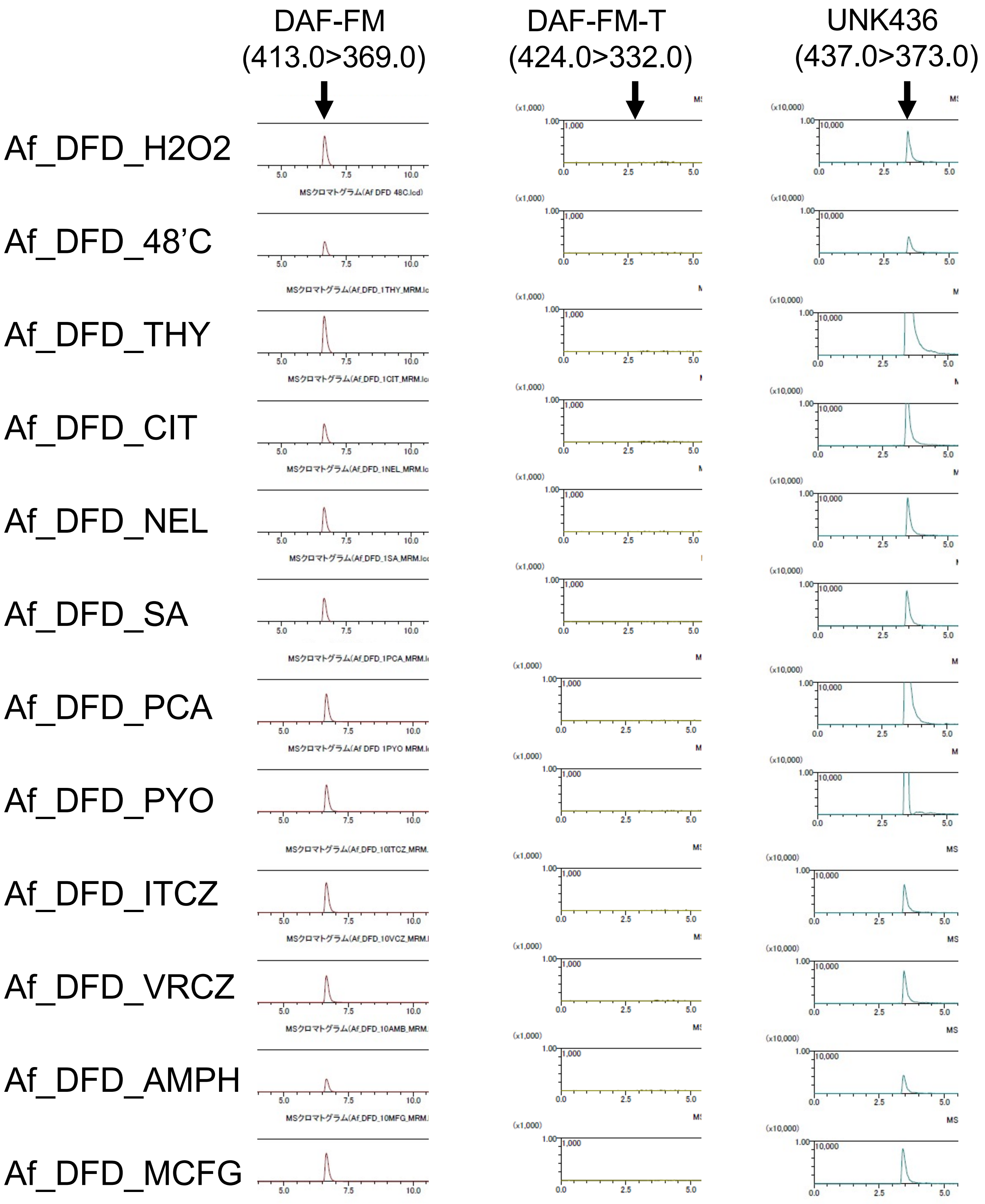

Supplementary Fig. S4. Detection of DAF-FM-T in *A. fumigatus* treated with DAF-FM DA and various stress factors. DAF-FM DA-treated cells were incubated with hydrogen peroxide (Af_DFD_H2O2), thymol (Af_DFD_THY), citral (Af_DFD_CIT), nerol (Af_DFD_NEL), salicylic acid (Af_DFD_SA), phenazine-1-carbonic acid (Af_DFD_PCA), pyocyanin (Af_DFD_PYO), itraconazole (Af_DFD_ITCZ), voriconazole (Af_DFD_VRCZ), amphotericin B (Af_DFD_AMPH), and micafungin (Af_DFD_MCFG). Each extract was applied to LC-MS/MS. DAF-FM, DAF-FM-T, and UNK436 was detected with fragments of 413.0>369.0, 424.0>332.0, and 437.0>373.0, respectively.
